# Brainstorm Pipeline Analysis of Resting-State Data From the Open MEG Archive

**DOI:** 10.3389/fnins.2019.00284

**Published:** 2019-04-05

**Authors:** Guiomar Niso, Francois Tadel, Elizabeth Bock, Martin Cousineau, Andrés Santos, Sylvain Baillet

**Affiliations:** ^1^McConnell Brain Imaging Centre, Montreal Neurological Institute, McGill University, Montreal, QC, Canada; ^2^Center for Biomedical Technology, Universidad Politécnica de Madrid, Madrid, Spain; ^3^Biomedical Image Technologies, ETSI Telecomunicación, Universidad Politécnica de Madrid, Madrid, Spain; ^4^Biomedical Research Networking Centre on Bioengineering, Biomaterials and Nanomedicine (CIBER-BBN), Madrid, Spain; ^5^Inserm U1216, Grenoble, France; ^6^Grenoble Institut des Neurosciences, Université Grenoble Alpes, Grenoble, France

**Keywords:** magnetoencephalography, resting-state, MEG-BIDS, power spectral density, reproducibility, analytical pipelines, open data, open science

## Abstract

We present a simple, reproducible analysis pipeline applied to resting-state magnetoencephalography (MEG) data from the Open MEG Archive (OMEGA). The data workflow was implemented with Brainstorm, which like OMEGA is free and openly accessible. The proposed pipeline produces group maps of ongoing brain activity decomposed in the typical frequency bands of electrophysiology. The procedure is presented as a technical proof of concept for streamlining a broader range and more sophisticated studies of resting-state electrophysiological data. It also features the recently introduced extension of the brain imaging data structure (BIDS) to MEG data, highlighting the scalability and generalizability of Brainstorm analytical pipelines to other, and potentially larger data volumes.

## Introduction

There is growing scientific interest in studying resting-state brain activity, where subjects do not perform a directed task or are not exposed to external stimuli. One of the many objectives of such studies is to understand the nature of regional brain activity and the mechanisms of network integration across the brain that are expressed in these task-free paradigms: e.g., resting state networks in fMRI (Damoiseaux et al., 2006), fMRI/EEG combined (Mantini et al., 2007), fMRI and MEG (de Pasquale et al., 2010), MEG (Brookes et al., 2011; Hillebrand et al., 2012; Florin and Baillet, 2015), resting-state activity alterations in diseases, such as mild cognitive impairments, Alzheimer’s disease (Fernández et al., 2006; Montez et al., 2009) and Parkinson’s Disease (Bosboom et al., 2006).

Here we provide the proof of technical concept for a basic data analysis pipeline designed with Brainstorm (Tadel et al., 2011) to extract group frequency-specific power analysis of regional source activity estimated with MEG, of healthy participants in the resting-state. We propose this pipeline as foundation to more sophisticated approaches and derivations, such as the extraction of resting-state network activity (e.g., Florin and Baillet, 2015). Brainstorm is a free, open-source application developed in Matlab and Java for multimodal electrophysiology and imaging. The resting-state MEG data was obtained from the Open MEG Archive, OMEGA, a free repository of MEG data (Niso et al., 2016). OMEGA is organized according to MEG-BIDS, a recent extension of the Brain Imaging Data Structure [BIDS^1^; (Niso et al., 2018)]. Brainstorm can directly import data from BIDS-organized data volumes. OMEGA contains multimodal data from 220 participants, for a total of 300 resting-state MEG recordings: 182 from healthy controls, 38 from patient volunteers (ADHD, chronic pain, etc.) as well as the anatomical T1-weighted MRI (T1w-MRI) volumes of all participants.

The present software pipeline is to demonstrate feasibility and reproducibility of the approach on the entirety of OMEGA, with generalizability to any other BIDS-organized data repository [see other MEG-BIDS data resources listed by Niso et al. (2018)]. The procedure produces maps of the regional power distribution of spontaneous brain activity in the typical frequency bands of electrophysiology: delta (2–4 Hz), theta (4–8 Hz), alpha (8–12 Hz), beta (15–30 Hz), gamma1 (30–80 Hz), and gamma2 (80–150 Hz).

We provide detailed descriptions of the main pipeline steps, with corresponding Matlab scripts distributed openly at GitHub as companions to this article, for easy replication (and extension) of the presented analyses and results^2^.

We refer the interested reader to (Tadel et al., 2011) for a detailed description of Brainstorm. Comprehensive tutorials for the application are available online^3^.

## Materials and Methods

The software, data and derivatives hereby produced require 22GB of disk space, on a conventional workstation or laptop. Brainstorm is freely available from http://neuroimage.usc.edu/brainstorm, with detailed installation instructions. Note that a Matlab license is not required, except for custom user scripting, which is not necessary to reproduce the analyses reported here.

### MEG and MRI Data From the Open MEG Archive

The OMEGA (Niso et al., 2016)^4^ is a collaborative effort to build and share a free MEG data repository. A unique aspect of OMEGA is that the resource is open-ended in the sense that its framework is designed for continued data aggregation, from interested investigators across the MEG community. In addition to MEG and T1w-MRI, OMEGA features demographic and questionnaire data. Basic demographic information include age, gender, handedness, and education. Additional non-identifying demographic characteristics include spoken languages, general health, alcohol consumption and smoking habits, sleep quality, chronic pain, and years of musical education and practice. For demonstration purposes, we used a subset of the data from 5 OMEGA healthy participants (2 females, 27+/- 5 y.o.), which is directly available from the open neuroimaging repository OpenNeuro.org^5^ 10.5 GB. To demonstrate generalizability beyond MEG-BIDS organized data, we also provide supplementary online material in the form of the same pipeline applied to MEG data from the Human Connectome Project (Larson-Prior et al., 2013); see Brainstorm tutorial^6^.

The 5 individual MEG datasets were collected from participants sitting upright, keeping their eyes open on a fixation cross for 5 min. No task instructions were provided except to refrain from producing eye movements and to remain awake. The data was acquired with a CTF MEG system at a single site (Montreal Neurological Institute, McGill University), after approval from the institutional research ethics board and from participants consenting to have their anonymized data shared via OMEGA. The MEG sensor array consisted of 275 axial gradiometers with 26 MEG reference sensors, located in a 3-layer magnetically shielded room. Sampling rate was 2400 Hz with a hardware anti-aliasing low-pass filter at 600 Hz. CTF 3rd-order gradient compensation was also applied. Bipolar electrocardiogram (ECG) and vertical and horizontal electrooculogram (EOG) data was collected on all subjects. Empty-room recordings (2-min duration or more) collected around each individual sessions were also retrieved from OMEGA to estimate the empirical noise statistics used in source modeling.

The individual head shapes, anatomical landmarks and fiducial points were collected during sessions and retrieved from the OMEGA sample dataset (^∗^.pos files). Fiducial points marked the location of three head position indicator (HPI) coils placed on the subject’s head: one on the forehead, one on the right, and the left mastoids. HPI coils are to track head position under the MEG helmet. Anatomical landmarks consisted of nasion and left/right preauricular points (NAS, LPA, and RPA, respectively) marked to facilitate geometrical co-registration between MEG sensor locations and structural MRI data. Finally, the locations of about 100 scalp points on hard parts of the head (away from soft tissues such as neck, cheeks, and mouth) were also digitized. They were used to refine cross-modal MEG/MRI co-registration, as explained below. All digitized points were collected using a Polhemus Fastrak device, driven by Brainstorm.

For anonymization purposes, T1w MRI images were defaced using free, open-source software (Face Masking^7^; Milchenko and Marcus, 2013). Scalp and cortical components were segmented and their envelopes were triangulated with Freesurfer 5.3 (Fischl, 2012), with default parameters. The co-registration procedure was facilitated by the convenient feature of BIDS that stores the anatomical landmarks and fiducials in a .json (JavaScript Object Notation) sidecar file of T1w MRI volumes. This information was read directly by Brainstorm and made the MEG/MRI co-registration process entirely automatic.

### A Note on MEG-BIDS

BIDS is a community-driven emerging standard for the organization of neuroimaging data. It was designed originally for structural and functional MRI (fMRI) (Gorgolewski et al., 2016). BIDS is based on a principled hierarchical folder structure, where folders contain data and extracted metadata of key study parameters documented in text-based human and machine readable formats.

We and collaborators recently contributed an extension of BIDS to MEG (Niso et al., 2018). Similar efforts are being pursued for scalp EEG and basic electrophysiology. MEG-BIDS facilitates data management and the design, sharing and transfer of analysis pipelines with well-distributed and -documented software applications [e.g., Brainstorm (Tadel et al., 2011), FieldTrip (Oostenveld et al., 2011), MNE (Gramfort et al., 2014), and SPM (Litvak et al., 2011)].

Brainstorm automatically imports (potentially large) MEG-BIDS datasets into its data management system, with the following folder/file organization for the OMEGA sample used in the present report:

**ds000247**/

• **sub-000X**/: Raw data for subject with ID code 000X.
  ○ **ses-0001**/: Here, only one session per subject.
    ◼ **sub-000X_ses-0001_scans.tsv**: tab-separated text file listing the MEG recordings and corresponding acquisition dates.
    ◼ **anat**/: Anatomical MRI scans for subject 000X (not used if /derivatives/freesurfer/sub-000X/ses-0001 is available).
      ◼ **sub-000X_T1w.nii.gz**: compressed T1w MRI data in Nifti format.
    ◼ **meg**: Raw MEG recordings.
      ◼ **sub-000X_ses-0001**_**task-rest_run-01_meg.ds**: Single run of MEG data.
• **derivatives**: Contains elements not considered as raw data.
    ◼ **freesurfer**: Output of the FreeSurfer segmentation pipeline for all participants.
      ◼ **sub-000X/ses-0001**: Output of FreeSurfer pipeline for subject 000X (session 0001).
• **sub-emptyroom**/: Empty-room recordings around the individual session dates.
  ○ **ses-XXX**/: Session of noise recordings (matched by date with the subjects’ recordings using the ^∗^_scans.tsv file).
    ◼ **sub-emptyroom_ses-XXX_scans.tsv**: tab-separated text file listing the MEG recordings and corresponding acquisition dates.
    ◼ **meg**/: Raw MEG empty-room recordings.
      ◼ **sub-000X_ses-XXX_task-noise_run-01_meg.ds.**

A (MEG-)BIDS validator is available online^8^ and is a convenient tool for verifying the integrity of a (MEG-)BIDS data distribution.

### Step 1: Import and Registration of Multimodal Data

**FIGURE 1:**
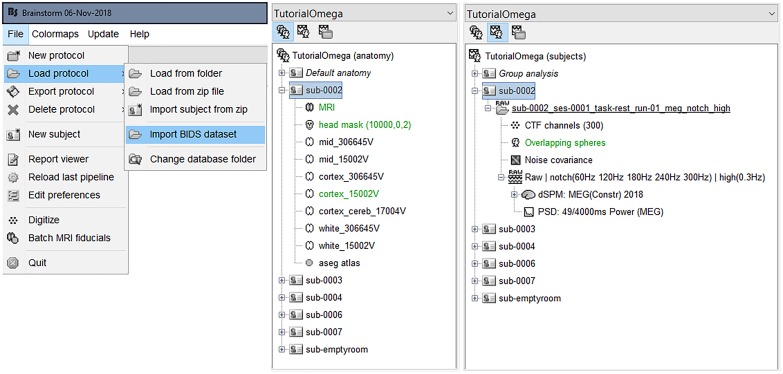
Brainstorm database entry created from the OMEGA sample dataset (ds000247). Left, one-step automatic importation of the MEG-BIDS OMEGA sample; Center, view of anatomy files; Right, view of MEG data files.

We created a new protocol (study) in Brainstorm selecting the menu item “*File > Create new protocol*” and named it “TutorialOmega” with the options: “*No, use individual anatomy*,” and *“No, use one channel file per condition*.” We then proceeded to importing the MEG-BIDS dataset directly, by selecting the menu item “*File > Load protocol > Import BIDS dataset > Select the folder sample_omega*.” We acknowledged all suggested default values during the import process e.g., the decimation of FreeSurfer cortical surface down to 15,000 vertices. Once this step was completed, the OMEGA sample of 5 participants was directly imported into Brainstorm’s data management system, including the associated empty-room recordings (Figure 1).

The coordinates of the NAS/LPA/RPA anatomical landmarks are contained in the MEG-BIDS data package, in both MEG and MRI spaces (^∗^_T1w.json files). Brainstorm uses these coordinates and the digitized head shape to automatically refine MEG-MRI co-registration using rigid-body transformations that minimize the distance of these points to the scalp surface automatically extracted from the structural MRI data by Brainstorm. This is performed during multimodal data importation and registration into Brainstorm’s database. Note that the MRI defacing procedure preserved the location of all fiducials points and of the scalp geometry. It is key to ascertain that the subjects’ head was well aligned under the MEG sensor array. To that purpose, we dragged and dropped the recordings from all subjects (excluding sub-emptyroom) into Brainstorm’s Process1 box, and clicked on the “Run” button. We then selected from the process menu “*Import anatomy > Remove head points, Z = 0*.” Finally, we added another process “*Import anatomy > Refine registration*.” To verify the quality of the registration procedure, we right-clicked on “*CTF channels > MRI registration > Check*” for each of the 5 participants.

### Step 2: Pre-processing of MEG Data

Some online signal processing was applied at the time of MEG acquisition (i.e., anti-aliasing low-pass filter below 600 Hz, CTF 3rd-order gradient compensation). Participant specific ^∗^_meg.json and ^∗^_channels.tsv files contain the details specific to each session and subject. Signal contamination from the environment (e.g., powerline, mechanical vibrations, etc.) or caused by participants (head and body movements, including breathing, eye blinks and saccades, heartbeats, muscle tension, and ferromagnetic prostheses) was evaluated and attenuated via the following good-practice preprocessing procedures (Gross et al., 2013).

We first reviewed the frequency contents of raw signals with power spectral density (PSD) estimates of MEG sensor signals. To do so, we switched to the functional view of the protocol (second button above Brainstorm’s database explorer). We then dragged and dropped all the data (including sub-emptyroom) into the Process1 box, and clicked on the “Run” button. Since CTF raw data are not usually saved as time-continuous but trial based, we selected the process “*Import recordings > Convert to continuous: Continuous*.” Then we estimated PSDs with “*Frequency > Power spectrum density (Welch): All file, 4s, 50% overlap, Individual*.” We followed the recommendations from Brainstorm’s online documentation to interpret PSD plots for assessing data quality^9^.

Next, we applied notch filters to eliminate powerline signal contamination at 60Hz and harmonics up to 300 Hz. We also applied a high-pass filter with a cutoff at 0.3 Hz to remove low-frequency fluctuations of no interest to the study. We verified the proper application of the frequency filters with a new set of PSDs. We processed the above files via the Process1 file selector and clicked on the “Run” button. We selected the process “*Pre-process > Notch filter: 60 120 180 240 300 Hz, Process the entire file at once*.” We added the process “*Pre-process > Band-pass filter: High-pass filter at 0.3 Hz, 60 dB, Process entire file*,” and added the process “*Frequency > Power spectrum density (Welch): Same options as before*” to the pipeline, before executing it by clicking “Run.”

**FIGURE 2:**
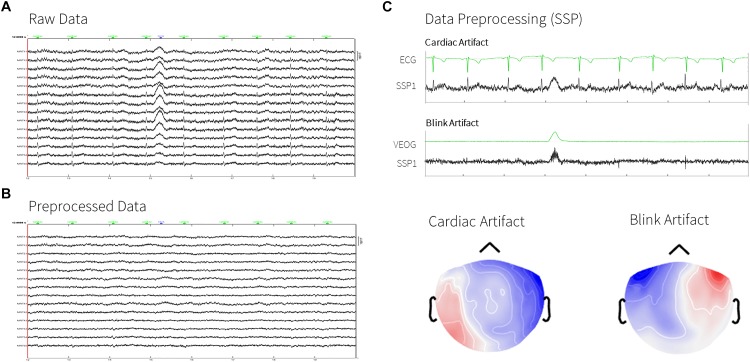
Removal of cardiac and blink artifacts. **(A)** Raw data from MEG sensors (8 s, subset of right temporal sensors, sub-0004, green dots indicating the detected heartbeats). **(B)** Processed data after artifacts removal. **(C)** ECG and VEO signals and their respectives SSP signals and topographies.

Most physiological signal contaminants are transient and potentially span a fairly large frequency range that overlaps with the frequency bands of interest to the study. We applied Signal-Space Projectors, SSPs (Gross et al., 2013) designed to attenuate physiological artifacts selectively. An SSP is produced by a principal component analysis of MEG traces around occurrences of signal artifacts of a given category (e.g., eye blinks, heartbeats). We used ECG and EOG traces to mark events of eye movements, blinks, and heartbeats. We then extracted epochs about these events to design the SSPs (see Figure 2). To this aim, we applied Brainstorm’s automatic processes for detecting and attenuating heartbeat and eye-blink signal contamination (for more details, please refer to Brainstorm’s tutorials^10^). This was performed automatically by dragging all recordings (from all subjects, excluding sub-emptyroom) into the Process1 box, and selecting the processes “*Events > Detect heartbeats: ECG, All file, cardiac*” and “*Events > Detect eye blinks: VEOG, All file, blink*.” We excluded signal portions where artifactual events of different categories occurred less than 250 ms from each other in time. This was derived automatically by selecting the process “*Events > Remove simultaneous: cardiac, when to close to blink, 250 ms*.” Then, we added the processes “*Artifacts > SSP: Heartbeats: cardiac, MEG, Use existing SSP*” and “*Artifacts > SSP: Eye Blinks: blink, MEG, Use existing SSP*” to this pipeline portion. To produce quality-control report logs of the analyses, we added the processes “*File > Snapshot: Sensor/MRI registration*” and “*File > Snapshot: SSP projectors*.”

We then reviewed the sensor topography of the selected SSPs and the MEG signal traces for all subjects to ascertain that the application of SSPs captured most of the MEG signal variance specific to heartbeat and blink contaminations (Figure 2). Heartbeats artifacts were correctly removed for all subjects using only the first SSP component (SSP1). Blink contamination was also properly removed for all subjects with the corresponding SSP1 component, except for sub-0003 and sub-0007. For these two subjects, additional blink SSPs were computed, after deleting the previous ones, this time using the *“Artifacts > SSP generic: All file, -200, 200 ms, 1.5–15 Hz, Use existing SSP, Average: One component only*” option. That way, instead of performing PCA over signal portions containing the detected blink events, a time-locked average of all blink events was computed to produce the corresponding SSP component. Other types of artifacts were also reviewed: we did not detect major contamination from saccades, except in sub-0002. For this latter participant, data contamination from eye saccades was attenuated using independent component analysis: we used the Brainstorm process *“Artifacts > ICA components: All file, 0 ms, 1–7Hz, 20 ICA components, HEOG, Use existing SSP, Infomax: EEGLAB/RunICA*” and selected the first ICA component to be removed from this participant’s recording. Muscle contamination was prominent in subject sub-0004: it was corrected using *“Artifacts > SSP generic: All file, 0 ms, 40–300 Hz, Use existing SSP, PCA*,” with selection of SSP1 and SSP2. We reviewed the first 100 s of data for detecting bad segments in all subjects, with no further data rejection performed.

The SSP/ICA cleaning procedure described here is an example designed for this specific dataset, with an emphasis on removing eye- and heart-related artifacts. For other types of experiments, acquisition devices, noise configurations or scientific questions, it might require adaptations (Gross et al., 2013). No preprocessing is a valid option: e.g., if the 1–4 Hz frequency band is of no interest in a study, correcting for eye blinks might not be necessary.

### Step 3: MEG Source Modeling

This section describes noise, head and source modeling to produce time-resolved maps of cortical currents in all participants (Baillet, 2017).

We first estimated empirical covariance statistics from the empty-room recordings, to characterize instrument and environmental noise. The noise covariance estimates were used for subsequent inclusion into the imaging estimator of distributed cortical currents (Baillet et al., 2001). In the Process1 box, we selected all the noise recordings (all the recordings in sub-emptyroom folder) and ran process “*Sources > Compute covariance: All file, Noise covariance, Copy to other folders, Copy to other subjects, Match by acquisition date*.”

This latter option (“*Match noise and subject recordings by acquisition date*”) reads the date of the session from the MEG-BIDS ^∗^_scans.tsv files to associate the noise covariance estimate to the participant data collected on the nearest session date.

We obtained an MEG forward model with the overlapping-spheres approach (Huang et al., 1999), automatically adjusted by Brainstorm to the participants’ scalp surface: we selected all resting-state recordings (all subjects, excluding sub-emptyroom) in Process1 box and ran the process “*Sources > Compute head model: Cortex surface, MEG = Overlapping spheres*.”

We then computed the imaging kernel of Brainstorm’s depth-weighted dynamic statistical parametric mapping constrained to the individual cortical surface of participants dSPM (Dale et al., 2000), running the process “*Sources > Compute sources [2018]: Kernel only, one per file, dSPM, constrained*.” When linearly applied to sensor data, this latter produces time-series estimates of cortical currents at each vertex location of the gray matter surface extracted from the individual MRI.

### Step 4: Frequency-Specific Brain Maps

We estimated the power of ongoing cortical activity in the typical frequency bands of electrophysiology: delta (2–4 Hz), theta (4–8 Hz), alpha (8–12 Hz), beta (15–30 Hz), gamma1 (30–80 Hz), and gamma2 (80–150 Hz). The present pipeline can be generalized to any other frequency band(s) of interest.

**FIGURE 3:**
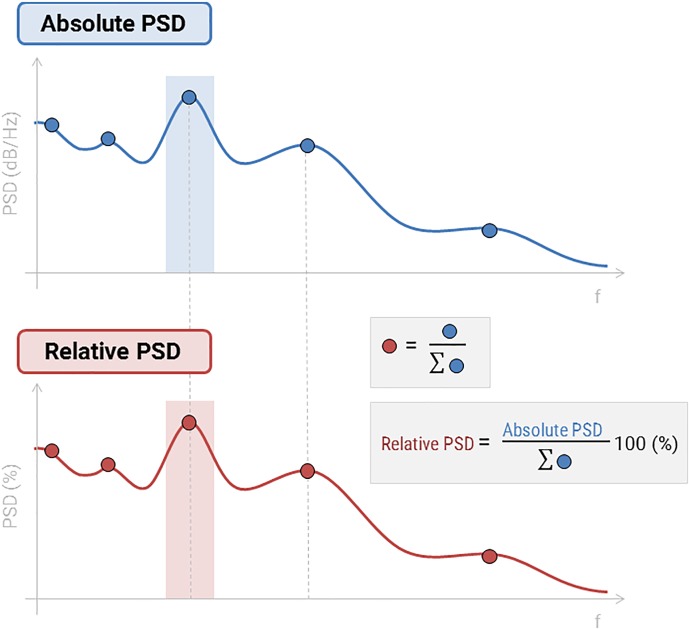
Absolute and relative PSD. Relative PSD values range between 0 and 1, indicating the contribution of the current frequency band to the total power in the signal.

We computed the PSD of all source time series for each participant. We then scaled the PSD values at each frequency bin relatively to the total power across the entire frequency spectrum: RelativePSD(f) = PSD(f)/Σ_i_[Total PSD(f_i_)], where f_i_’s are the individual frequencies from the original (absolute) PSD (Figure 3). This procedure is to standardize PSD values across brain regions and participants.

We used Welch’s method for estimating PSDs over the first 100 s of each MEG recording, with 4-s sliding Hamming windows overlapping at 50%. We dragged all resting-state recordings (i.e., recordings which include ^∗^_task-rest in their names) in the Process1 box, and clicked the “Process sources” button to select the process *“Frequency > Power spectrum density (Welch): [0,100 s], Window = 4 s, 50% overlap, Group in frequency bands (use the default frequency bands), Save individual PSD values*.” This latter process regroups PSD in frequency bands, averaging PSD bins within each band of interest. We then added the processing step “*Standardize > Spectrum normalization: Relative power (divide by total power)*,” which derives at each source location and for each frequency band the ratio of how much the signal in the frequency band contributes to the total power of the source signal.

**FIGURE 4:**
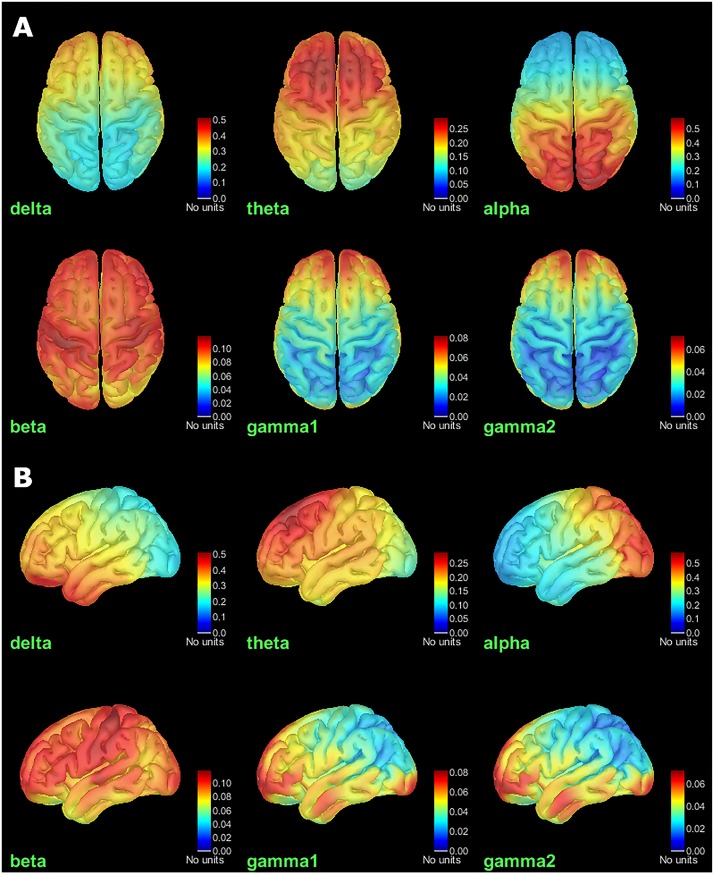
Group average of relative PSD maps for all tested frequency bands. **(A)** Top View. **(B)** Left view. Values range between 0 and 1, indicating the power of cortical signals relatively to the total signal power across the frequency spectrum.

To produce a group-average PSD map, we projected individual results onto a common brain template MNI ICBM152 (Fonov et al., 2009). Brainstorm template projection aligns the cortical curvature maps in spherical topology, following the approach implemented in FreeSurfer (Tadel et al., 2019). We then applied a surface smoothing kernel on each original map, by assembling together the individual processes “*Sources > Project on default anatomy: Cortex*” and “*Sources > Spatial smoothing: FWHM = 3 mm, Overwrite*.” This latter step was to smooth individual cortical maps using a circularly symmetric Gaussian surface kernel with a full width half maximum (FWHM) size of 3 mm. This process relies on the function ‘SurfStatSmooth’, implemented in SurfStat (Worsley et al., 2009). Finally, we produced the group average of PSD maps, dropping all the projected individual files from the Group analysis folder in Process1 box, and clicking on the button “Process time-freq” to run the process “*Average > Average files: Everything, Arithmetic average, Do not match signals.*” The results can be displayed by double-clicking on the average result file entry in the Brainstorm data tree. To generate Figure 4, we right-clicked on the figure and selected “*Snapshot > Frequency contact sheet*.”

A similar analysis can be performed at the sensor level: We first computed the total and relative PSD by frequency band of the continuous recordings for each subject. We dragged all recordings (task-rest) in the Process1 box, and clicked the “Process signals” button to select the process *“Frequency > Power spectrum density (Welch): [0,100 s], Window = 4 s, 50% overlap, Group in frequency bands (use the default frequency bands), Save individual PSD values*” and added the process “*Standardize > Spectrum normalization: Relative power (divide by total power)*.” We then computed the group average of the resulting individual PSD sensor data. We dropped the individual results for each subject in the Group analysis folder in Process1 box, and clicked on the button “Process time-freq” to run the process “*Average > Average files: Everything, Arithmetic average, Do not match signals*.” The results can also be displayed by double-clicking on the average result file entry in the Brainstorm data tree, and to produce Figure 6, we right-clicked on the figure and selected “*Snapshot > Frequency contact sheet*.”

## Results

We obtained brain maps of relative power for each source and each frequency band of interest (Figure 4). At each vertex of the cortical surface, the value reported represents the fraction (between 0 and 1) of signal power in current frequency band with respect to the entire PSD across the frequency spectrum.

The healthy population PSD maps obtained are consistent with results previously reported by Niso et al. (2016) and in the literature, mainly reported at the sensor level (Ishii et al., 1999), alpha-gamma coupling (Roux et al., 2013). We found stronger activity in the delta band over the frontorobital regions and anterior temporal poles. Theta band activity was distributed bilaterally over the frontal lobe. Alpha activity was dominant over parieto-occipital regions, and beta-band relative power was stronger over the pre and post-central lobules. Finally, low and high gamma ongoing activity was dominant over pre-frontal and occipital regions.

We questioned whether the observed concentrations of delta and low/high gamma activity could be of artifactual origins, still remaining after the signal corrections applied. For instance, residual contamination from large eye movements and blinks would explain stronger delta activity around the eye sockets. Similarly, the inferior occipital regions where gamma activity was the strongest could be related to muscular tension in the neck.

**FIGURE 5:**
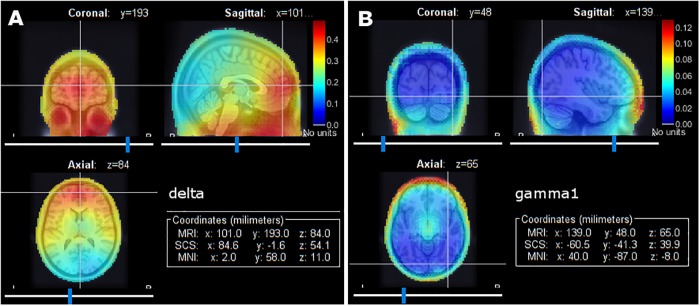
Group average of relative signal power using a 3-D grid source model in full head volume. **(A)** Relative signal power in the delta band. **(B)** Relative signal power in the gamma1 band.

To clarify these aspects, we derived another source model of the data, using a uniform (not cortically constrained) 3-D dipole grid across the entire head volume (see online tutorial^11^). The new model confirmed some concentration of delta and gamma power over the eye sockets and upper neck muscles, although not critical (Figure 5). This rapid quality control procedure is to encourage users to proceed with caution when interpreting source maps and highlights the importance of careful artifact rejection to ascertain the neural origins of the signals.

**FIGURE 6:**
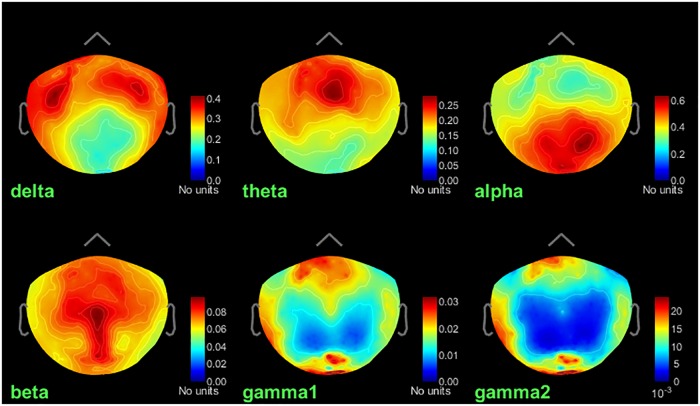
Group average of relative PSD of sensor data for the frequency bands. Top sensor view. Values range between 0 and 1, indicating the power of cortical signals relatively to the total signal power across the frequency spectrum.

Similar derivations can be produced at the sensor level. There are caveats to averaging sensor data between participants: in source space, individual data was registered to a common brain template, and differences in individual head positions across participants was accounted for by source modeling. With sensor data though, individual head positions are not systematically accounted for with respect to the rigid MEG sensor array. Hence, the same sensor does not necessarily pick up equivalent brain regions across participants. For this reason, the sensor results shown Figure 6 are essentially illustrative and to qualitatively assess consistency with source maps.

## Conclusion

We reported a simple, reproducible analysis pipeline, with Brainstorm operating on resting-state MEG BIDS data retrieved from the OMEGA. We illustrated the approach with 5 data volumes hosted on OpenNeuro.org. The pipeline details the group analysis performed, including basic steps for preprocessing, source reconstruction and the estimation of brain, and sensor distributed relative PSD group statistics in the typical frequency bands of electrophysiology.

The analysis pipeline presented here is fully reproducible via the following steps. Importantly, we provide a Matlab script as part of the standard Brainstorm distribution (tutorial_omega.m^12^) that runs all steps at once automatically.

(1) Download the data^13^, and unzip it in a folder (let the directory be *BidsDir*); it requires about 10.5GB of free storage space.
(2) Note that getting the data from a web browser as a single zip file did not work well at time of submission, another more reliable solution using the Amazon AWS CLI software is described on the Brainstorm online tutorials^14^.
(3) Download and install Brainstorm^15^. In general, we recommend getting the most up-to-date version available from the Brainstorm website, however, for the strict reproducibility of the results presented in this article, we uploaded a development snapshot from November 15th, 2018, on the Zenodo website^16^.
(4) Launch Brainstorm, set the software’s database folder as explained in Brainstorm’s installation instructions.
(5) Close Brainstorm.
(6) In the Matlab command window, type: tutorial_omega (*BidsDir*).
(7) This will run the full pipeline on the downloaded data, which requires another 11.5GB of additional free storage space.
(8) Execution time is typically up to 5 h on a conventional workstation.

This pipeline can be applied to other datasets, for instance with EEG data or with other source modeling approaches, as long as the original data is BIDS-organised.

## Ethics Statement

The article we submitted did not involve any original data collection. The data used is the one presented in these publications: https://www.sciencedirect.com/science/article/pii/S1053811915003183 and https://www.nature.com/articles/sdata2018110. Please refer to the details therein.

## Author Contributions

GN, FT, EB, and SB designed the analysis pipeline. GN deployed the efforts for BIDS data standardization. FT and MC developed the software and documentation. GN and SB wrote the manuscript. All authors contributed to manuscript revision.

## Conflict of Interest Statement

The authors declare that the research was conducted in the absence of any commercial or financial relationships that could be construed as a potential conflict of interest.

## Footnotes

1 http://bids.neuroimaging.io

2 https://github.com/brainstorm-tools/brainstorm3/blob/master/toolbox/script/tutorial_omega.m

3 http://neuroimage.usc.edu/brainstorm/Tutorials

4 https://www.mcgill.ca/bic/resources/omega

5 https://openneuro.org/datasets/ds000247/

6 https://neuroimage.usc.edu/brainstorm/Tutorials/HCP-MEG

7 http://nrg.wustl.edu/software/face-masking/

8 http://bids-standard.github.io/bids-validator/

9 http://neuroimage.usc.edu/brainstorm/Tutorials/ArtifactsFilter#Interpretation_of_the_PSD

10 https://neuroimage.usc.edu/brainstorm/Tutorials/

11 http://neuroimage.usc.edu/brainstorm/Tutorials/CoregisterSubjects#Volume_source_model

12 https://github.com/brainstorm-tools/brainstorm3/blob/master/toolbox/script/tutorial_omega.m

13 https://openneuro.org/datasets/ds000247/

14 https://neuroimage.usc.edu/brainstorm/Tutorials/VisualSingle#Download_and_installation

15 http://neuroimage.usc.edu/brainstorm/Installation

16 https://zenodo.org/record/1489533

## References

[B1] Baillet S. (2017). “Magnetoencephalography for brain electrophysiology and imaging.” *Nat. Neurosci.* 20 327–339. 10.1038/nn.4504 28230841

[B2] Baillet S. Mosher J. C. Leahy R. M. (2001). Electromagnetic brain mapping. *IEEE Signal Process. Mag.* 18 14–30. 10.1109/79.962275

[B3] Bosboom J. L. Stoffers D. Stam C. J. van Dijk B. W. Verbunt J. Berendse H. W. (2006). Resting state oscillatory brain dynamics in parkinson’s disease: an meg study. *Clin. Neurophysiol.* 117 2521–2531. 10.1016/j.clinph.2006.06.720 16997626

[B4] Brookes M. J. Woolrich M. Luckhoo H. Price D. Hale J. R. Stephenson M. C. (2011). “Investigating the electrophysiological basis of resting state networks using magnetoencephalography.” *Proc. Natl. Acad. Sci. U.S.A.* 108 16783–16788. 10.1073/pnas.1112685108 21930901 PMC3189080

[B5] Dale A. M. Liu A. K. Fischl B. R. Buckner R. L. Belliveau J. W. Lewine J. D. (2000). Dynamic statistical parametric mapping: combining fMRI and MEG for high-resolution imaging of cortical activity. *Neuron* 26 55–67. 10.1016/S0896-6273(00)81138-1 10798392

[B6] Damoiseaux J. S. Rombouts S. A. Barkhof F. Scheltens P. Stam C. J. Smith S. M. (2006). “Consistent resting-state networks across healthy subjects.” *Proc. Natl. Acad. Sci. U.S.A.* 103 13848–13853. 10.1073/pnas.0601417103 16945915 PMC1564249

[B7] de Pasquale F. Della Penna S. Snyder A. Z. Lewis C. Mantini D. Marzetti L. (2010). “Temporal Dynamics of spontaneous MEG activity in brain networks”. *Proc. Natl. Acad. Sci. U.S.A.* 107 6040–6045. 10.1073/pnas.0913863107 20304792 PMC2851876

[B8] Fernández A. Hornero R. Mayo A. Poza J. Gil-Gregorio P. Ortiz T. (2006). MEG spectral profile in alzheimer’s disease and mild cognitive impairment. *Clin. Neurophysiol.* 117 306–314. 10.1016/j.clinph.2005.10.017 16386951

[B9] Fischl B. (2012). FreeSurfer. *NeuroImage* 62 774–781. 10.1016/j.neuroimage.2012.01.021 22248573 PMC3685476

[B10] Florin E. Baillet S. (2015). The brain’s resting-state activity is shaped by synchronized cross-frequency coupling of neural oscillations. *NeuroImage* 111 26–35. 10.1016/j.neuroimage.2015.01.054 25680519 PMC4387013

[B11] Fonov V. S. Evans A. C. McKinstry R. C. Almli C. R. Collins D. L. (2009). Unbiased nonlinear average age-appropriate brain templates from birth to adulthood. *NeuroImage* 47:S102 10.1016/S1053-8119(09)70884-5

[B12] Gorgolewski K. J. Auer T. Calhoun V. D. Craddock R. C. Das S. Duff E. P. (2016). The brain imaging data structure, a format for organizing and describing outputs of neuroimaging experiments. *Sci. Data* 3:160044. 10.1038/sdata.2016.44 27326542 PMC4978148

[B13] Gramfort A. Luessi M. Larson E. Engemann D. A. Strohmeier D. Brodbeck C. (2014). MNE software for processing MEG and EEG Data. *NeuroImage* 86 446–460. 10.1016/j.neuroimage.2013.10.027 24161808 PMC3930851

[B14] Gross J. Baillet S. Barnes G. R. Henson R. N. Hillebrand A. Jensen O. (2013). Good practice for conducting and reporting MEG research. *NeuroImage* 65 349–363. 10.1016/j.neuroimage.2012.10.001 23046981 PMC3925794

[B15] Hillebrand A. Barnes G. R. Bosboom J. L. Berendse H. W. Stam C. J. (2012). Frequency-dependent functional connectivity within resting-state networks: an atlas-based MEG beamformer solution. *NeuroImage* 59 3909–3921. 10.1016/j.neuroimage.2011.11.005 22122866 PMC3382730

[B16] Huang M. X. Mosher J. C. Leahy R. M. (1999). A sensor-weighted overlapping-sphere head model and exhaustive head model comparison for MEG. *Phys. Med. Biol.* 44 423–440. 10.1088/0031-9155/44/2/010 10070792

[B17] Ishii R. Shinosaki K. Ukai S. Inouye T. Ishihara T. Yoshimine T. (1999). Medial prefrontal cortex generates frontal midline theta rhythm. *Neuroreport* 10 675–679. 10.1097/00001756-199903170-00003 10208529

[B18] Larson-Prior L. J. Oostenveld R. Della Penna S. Michalareas G. Prior F. Babajani-Feremi A. (2013). Adding dynamics to the human connectome project with MEG. *NeuroImage* 80 190–201. 10.1016/j.neuroimage.2013.05.056 23702419 PMC3784249

[B19] Litvak V. Mattout J. Kiebel S. Phillips C. Henson R. Kilner J. (2011). EEG and MEG data analysis in SPM8. *Comput. Intel. Neurosci.* 2011:852961. 10.1155/2011/852961 21437221 PMC3061292

[B20] Mantini D. Perrucci M. G. Del Gratta C. Romani G. L. Corbetta M. (2007). Electrophysiological signatures of resting state networks in the human brain. *Proc. Natl. Acad. Sci. U.S.A.* 104 13170–13175. 10.1073/pnas.0700668104 17670949 PMC1941820

[B21] Milchenko M. Marcus D. (2013). Obscuring surface anatomy in volumetric imaging data. *Neuroinformatics* 11 65–75. 10.1007/s12021-012-9160-3 22968671 PMC3538950

[B22] Montez T. Poil S. S. Jones B. F. Manshanden I. Verbunt J. P. van Dijk B. W. (2009). Altered temporal correlations in parietal alpha and prefrontal theta oscillations in early-stage alzheimer disease. *Proc. Natl. Acad. Sci. U.S.A.* 106 1614–1619. 10.1073/pnas.0811699106 19164579 PMC2635782

[B23] Niso G. Gorgolewski K. J. Bock E. Brooks T. L. Flandin G. Gramfort A. (2018). MEG-BIDS, the brain imaging data structure extended to magnetoencephalography. *Sci. Data* 5:180110. 10.1038/sdata.2018.110 29917016 PMC6007085

[B24] Niso G. Rogers C. Moreau J. T. Chen L. Y. Madjar C. Das S. (2016). OMEGA: the open MEG archive. *NeuroImage* 124(Pt B), 1182–1187. 10.1016/j.neuroimage.2015.04.028 25896932

[B25] Oostenveld R. Fries P. Maris E. Schoffelen J. M. (2011). FieldTrip: open source software for advanced analysis of MEG, EEG, and invasive electrophysiological data. *Comput. Intel. Neurosci.* 2011:156869. 10.1155/2011/156869 21253357 PMC3021840

[B26] Roux F. Wibral M. Singer W. Aru J. Uhlhaas P. J. (2013). The phase of thalamic alpha activity modulates cortical gamma-band activity: evidence from resting-state MEG recordings. *J. Neurosci.* 33 17827–17835. 10.1523/JNEUROSCI.5778-12.2013 24198372 PMC3818555

[B27] Tadel F. Baillet S. Mosher J. C. Pantazis D. Leahy R. M. (2011). Brainstorm: a user-friendly application for MEG/EEG analysis. *Comput. Intel. Neurosci.* 2011:879716. 10.1155/2011/879716 21584256 PMC3090754

[B28] Tadel F. Bock E. Niso G. Mosher J. C. Cousineau M. Pantazis D. (2019). MEG/EEG group analysis with brainstorm. *Front. Neurosci.* 13:76. 10.3389/fnins.2019.00076 30804744 PMC6378958

[B29] Worsley K. J. Taylor J. E. Carbonell F. Chung M. K. Duerden E. Bernhardt B. (2009). SurfStat: a matlab toolbox for the statistical analysis of univariate and multivariate surface and volumetric data using linear mixed effects models and random field theory. *NeuroImage* 47:S102 10.1016/S1053-8119(09)70882-1

